# Increased growth rates of stream salamanders following forest harvesting

**DOI:** 10.1002/ece3.8238

**Published:** 2021-10-24

**Authors:** Jacquelyn C. Guzy, Brian J. Halstead, Kelly M. Halloran, Jessica A. Homyack, John D. Willson

**Affiliations:** ^1^ Wetland and Aquatic Research Center U.S. Geological Survey Davie Florida USA; ^2^ Department of Biological Sciences University of Arkansas Fayetteville Arkansas USA; ^3^ Western Ecological Research Center U.S. Geological Survey Dixon California USA; ^4^ Strategy and Technology Weyerhaeuser Company Centralia Washington USA

**Keywords:** Arkansas, before–after control–impact, capture–mark–recapture, *Desmognathus brimleyorum*, interior highlands, Ouachita dusky salamander, Ouachita Mountains, streamside management zone

## Abstract

Timber harvesting can influence headwater streams by altering stream productivity, with cascading effects on the food web and predators within, including stream salamanders. Although studies have examined shifts in salamander occupancy or abundance following timber harvest, few examine sublethal effects such as changes in growth and demography. To examine the effect of upland harvesting on growth of the stream‐associated Ouachita dusky salamander (*Desmognathus brimleyorum*), we used capture–mark–recapture over three years at three headwater streams embedded in intensely managed pine forests in west‐central Arkansas. The pine stands surrounding two of the streams were harvested, with retention of a 14‐ and 21‐m‐wide forested stream buffer on each side of the stream, whereas the third stream was an unharvested control. At the two treatment sites, measurements of newly metamorphosed salamanders were on average 4.0 and 5.7 mm larger post‐harvest compared with pre‐harvest. We next assessed the influence of timber harvest on growth of post‐metamorphic salamanders with a hierarchical von Bertalanffy growth model that included an effect of harvest on growth rate. Using measurements from 839 individual *D*. *brimleyorum* recaptured between 1 and 6 times (total captures, *n* = 1229), we found growth rates to be 40% higher post‐harvest. Our study is among the first to examine responses of individual stream salamanders to timber harvesting, and we discuss mechanisms that may be responsible for observed shifts in growth. Our results suggest timber harvest that includes retention of a riparian buffer (i.e., streamside management zone) may have short‐term positive effects on juvenile stream salamander growth, potentially offsetting negative sublethal effects associated with harvest.

## Graphical abstract

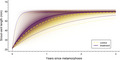

## 1 INTRODUCTION

Headwater streams are strongly influenced by harvesting of timber within the surrounding watershed (Webster et al., 1992). The most evident direct effect of harvesting on forest streams is the removal of shading vegetation, which alters stream microclimates (Olson et al., 2007) and results in both increased average stream temperatures (Reiter et al., 2015) and reduction in allochthonous inputs (i.e., leaf litter; Webster & Waide, 1982). Consequently, following harvest, streams are less light‐limited and filamentous green algae often increase in abundance (Lowe et al., 1986), increasing total primary production (Webster et al., 1983). Accompanying this shift in the stream energy base is often a switch in dominant benthic invertebrates (Gurtz & Wallace, 1984; Wallace, 1988; Wallace & Gurtz, 1986) from shredders to scrapers and collectors that feed on algae (Wallace et al., 1997; Webster et al., 1992). Other potential effects of forest harvesting include short‐term increases in stream flow with less evapotranspiration and potential alterations to nutrient processing (i.e., nitrogen and phosphorus loss; Webster et al., 1992).

Stream salamanders are a primary vertebrate predator within headwater stream systems where they consume both aquatic and terrestrial invertebrate detritivores (Johnson & Wallace, 2005; Southerland et al., 2004) and can attain extremely high densities and biomass (e.g., 11,294 salamanders/ha; Peterman et al., 2008). As such, they can exert direct and indirect biotic control of prey species and influence ecosystem processes along grazer and detrital pathways (reviewed in Davic & Welsh, 2004). These trophic associations may ultimately influence the breakdown of leaf litter and transfer of nutrients (Davic & Welsh, 2004; Walker et al., 2018; Wyman, 1998) and, importantly, can be influenced by forest harvesting, particularly when allochthonous inputs of leaf litter are reduced (Johnson & Wallace, 2005; Wallace et al., 1997).

Numerous studies across North America report that salamander populations decline for a period of time after timber harvesting (e.g., Ash, 1997; Connette & Semlitsch, 2015; Herbeck & Larsen, 1999; Petranka et al., 1994; Reichenbach & Sattler, 2007). However, recent research has suggested that forestry best management practices (BMPs), specifically implementation of riparian buffers (referred to as streamside management zones/SMZs in some U.S. regions; Lee et al., 2004), may ameliorate negative effects on salamander movement (Johnston & Frid, 2002), abundance (Halloran et al., 2021; Maigret et al., 2014; Perkins & Hunter, 2006; Peterman & Semlitsch, 2009), and species richness and occupancy (Guzy et al., 2019; Kroll et al., 2008). Yet, it is unclear whether harvesting can affect fitness surrogates such as stream salamander growth and reproduction. Little research has examined salamander response to forestry activities at the individual level (i.e., mark–recapture approaches; but see Cecala et al., 2014; Chazal & Niewiarowski, 1998; Connette & Semlitsch, 2015), and to our knowledge, none have examined changes in individual salamander growth rates in response to timber harvesting, either for woodland or for stream‐associated salamanders. Although a few studies have explored the influence of forest management on endpoints such as body condition (Hocking et al., 2013; Homyack et al., 2011; Karraker & Welsh, 2006), examining growth at the individual level is necessary to address alternative explanations for changes in population demography such as size‐biased mortality.

Measuring growth as a potential response to harvest is particularly important because body size influences survival and fecundity of salamanders and thus contributes to individual fitness and population growth (Hernández‐Pacheco et al., 2021; Tilley, 1968). Energetic requirements of salamanders may vary with differences in the thermal environment of harvested areas (Homyack et al., 2011), influencing metabolic rates, growth, and ultimately body size. Similarly, because stream salamander growth has been correlated with prey biomass (Huntsman et al., 2011; Johnson & Wallace, 2005), changes in the stream invertebrate community caused by harvesting within the watershed may influence salamander growth and body size (e.g., Bumpers et al., 2017).

In this study, we used intensive capture–mark–recapture at three streams to examine the effect of upland forest harvesting on growth of a stream salamander species, the Ouachita dusky salamander (*Desmognathus brimleyorum*). To reduce the influence of stochastic differences among sites and years, we used a before–after control–impact (BACI) design, which allowed for comparisons within the same sites before and after harvest, and comparisons with a designated control site through time. Although our streams contained riparian buffers (SMZs), we predicted that stream salamander growth rate would be faster and body condition would be higher immediately post‐harvest, due to short‐term increased productivity resulting from canopy reduction or potential nutrient increases.

## 2 METHODS

### 2.1 Study species

*Desmognathus brimleyorum* occurs in west‐central Arkansas and southwest Oklahoma (Means, 1999) and is one of the least studied *Desmognathus* species (Petranka, 1998). Oviposition typically occurs during summer months (Trauth et al., 1988), and has been reported to peak in late June and early July in the Cossatot Mountains (Means, 1975), but may occur anytime from March to September (Trauth et al., 1990). The larval period has not been well established but is thought to last approximately 10 months to a year (Means, 1974; Trauth et al., 1990). Our observations of 148 individuals indicate that metamorphs range from ~20–30 mm SVL. Within west‐central Arkansas, average length for adults has been reported from the Cossatot Mountains as 64 mm SVL (females) and 71 mm SVL (males) (Means, 1999). Here, we considered individuals >45 mm SVL at time of capture as adults, and based on 349 individuals, average adult length for our study area is 56 mm (SD 7.6 mm); we observed a maximum of 80 and 79 mm SVL for females and males, respectively. Based on our mark–recapture dataset, individuals may live to at least seven years. Females have been reported to reproduce at >63 mm SVL (Means, 1974; Trauth et al., 1990), and in our study, gravid females were on average 61 mm (*n* = 144), although we observed gravid females as small as 49–55 mm SVL (*n* = 10). In a concurrent study, Halloran et al. (2021) reported average net movement (i.e., distance between furthest upstream and furthest downstream capture locations) to be less than 20 m with a slight upstream movement bias, although a few individuals moved up to 164 m within the stream. However, movement was increased following upland clear‐cut timber harvest; 35% of 1030 *D*. *brimleyorum* at the control site had a net movement of 4 m or less, but at treatment sites 19% of the 1423 individuals had net in‐stream movements less than 4 m between pre‐harvest and post‐harvest surveys (Halloran et al., 2021).

### 2.2 Study sites

**FIGURE 1:**
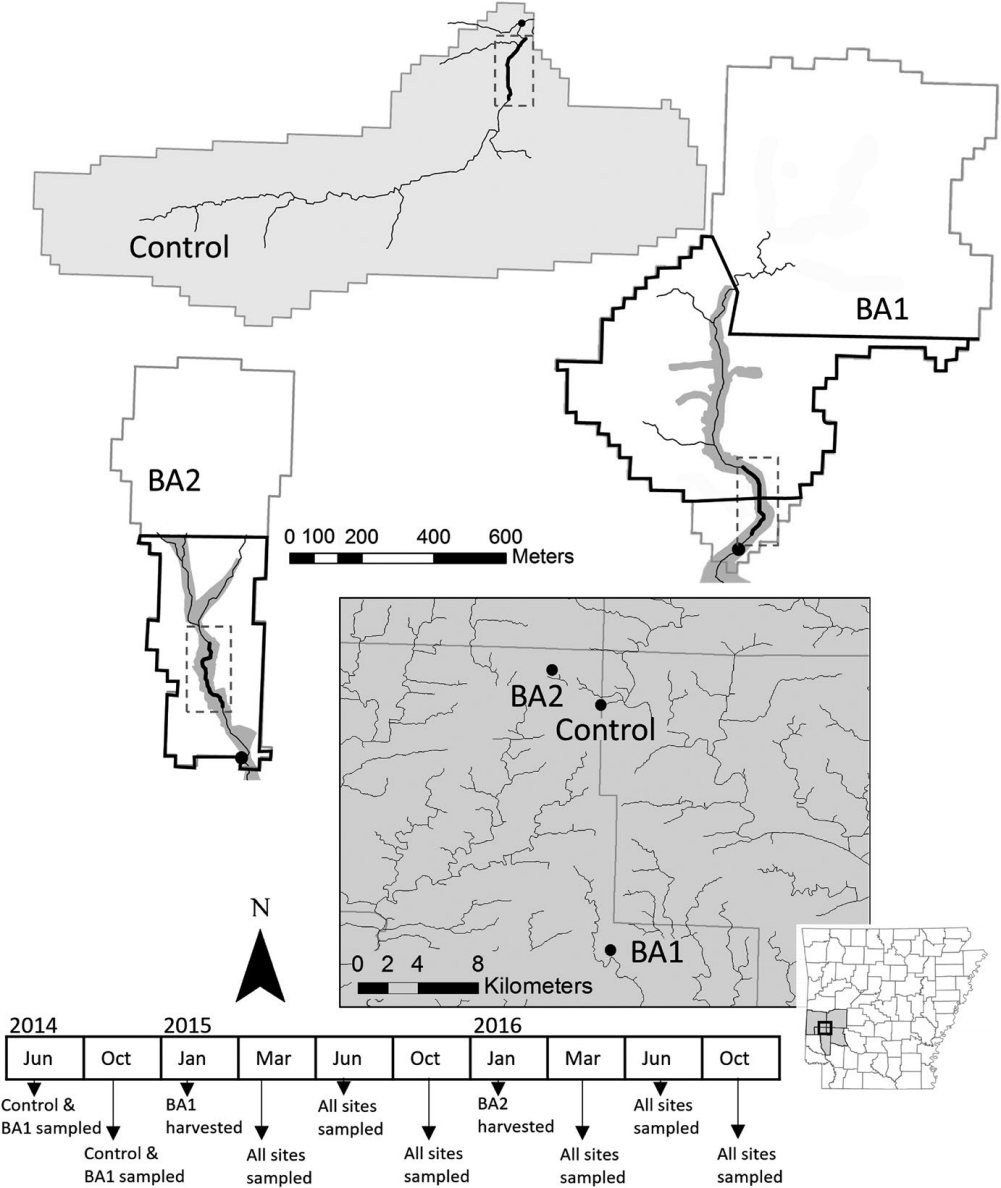
Location of study sites in northeast Howard County, Arkansas, USA, and timeline of timber harvest and sampling schedule. Stream watersheds are outlined in gray. Approximate streamside management zone (SMZ) boundary for treatment sites (BA1, BA2) is shaded in gray. Bold black lines enclosed in a dashed box indicate the 200‐m sampling transect where salamanders were sampled. At before–after sites, the harvested section of the watershed is outlined in black

This study was conducted in northeast Howard County, in west‐central Arkansas, USA, within 7–23 km of the Cossatot Mountains, in the southernmost subdivision of the Ouachita Mountains (Figure 1), and occurred within even‐aged loblolly pine (*Pinus taeda*) forest managed by Weyerhaeuser Company. To assess the influence of timber harvest on the stream‐dwelling Ouachita dusky salamander (*Desmognathus brimleyorum*), we selected three 1^st^‐order, intermittent headwater streams based on similar size, morphology, and silvicultural history. Sites were located within 16 km of each other in the Little Missouri River Watershed, with elevations ranging from 190 to 300 m above sea level. Each stream drained a small watershed (0.41–1.15 km^2^) within a mature (29–35 years old) loblolly pine stand.

### 2.3 Study design

We used a BACI study design to examine the effects of timber harvest on salamander growth. Specifically, we conducted intensive capture–mark–recapture of salamanders at one “control” (unharvested) site and two before–after sites (hereafter “BA1” and “BA2”) that were clear‐cut‐harvested during the study, with an SMZ retained along each stream. We conducted salamander surveys at the control and BA1 sites from 2014 to 2016, during March, June, and October of each year; during each month, each site was sampled three times (approximately one week apart; Figure 1). The same survey schedule was implemented for BA2; however, surveys began one year later in March 2015. At each site, we established a 200 m stream transect at the most downstream section of each stream. The BA1 and BA2 sites were clear‐cut‐harvested in January 2015 and January 2016, respectively, with a 28‐m and a 42‐m SMZ retained along the length of the stream (Figure 1). In concordance with state BMPs, some overstory pine trees were harvested from the SMZ of BA1 to promote hardwood regeneration. The SMZ at BA2 along with the riparian forest surrounding the control site was comprised of an oak‐hickory (*Quercus* and *Carya* spp.) overstory with a cedar (*Juniperus virginiana*) and holly (*Ilex opaca*) understory. The SMZ of the BA1 site was dominated by loblolly pine in the overstory and holly, musclewood (*Carpinus caroliniana*), and hophornbeam (*Ostrya virginiana*) in the understory. Based on 16–28 measures of canopy cover taken with a concave spherical densiometer (Lemmon, 1956) in the center of each stream transect before and after harvest after leaf‐out (i.e., May), canopy cover decreased by 18% (100 vs. 82%) at BA1 and by 24% (99% vs. 75%) at BA2 following harvesting, but remained constant at 98.9%–100% at the control site.

### 2.4 Field methods

Each salamander survey began approximately one hour after sunset and consisted of a thorough visual search (i.e., turning over rocks and debris) of the streambed for the length of each 200‐m transect. Post‐metamorphic *Desmognathus brimleyorum* were captured using dip‐nets and placed in separate containers, and each individual's location was marked with a flag. The following day, we processed captured salamanders in the laboratory by anesthetizing each individual with a solution of 1 g Orajel™‐20% benzocaine/1 L of de‐chlorinated tap water (Cecala et al., 2007) and recording body metrics using a digital scale and calipers [i.e., mass (g), total length (mm), snout–vent length (SVL; mm)]. All salamanders were anesthetized regardless of recapture status to allow for precise measurements of length and mass. Following measurement, each newly captured individual was given a unique identification mark using a subcutaneous injection of visible implant elastomer (VIE; Northwest Marine Technologies; Grant, 2008). We ventrally marked individuals using a combination of four colors (pink, orange, blue, and yellow) and 6 marking locations (posterior to each limb and anterior to each hind limb) with a 0.5‐ml Micro‐Fine™ insulin syringe (28‐gauge/0.35 mm). To ensure reliable identification, each salamander was marked at a minimum of two positions using at least two colors. Any recently metamorphosed individuals (less than a year since metamorphosis; <45 mm SVL) were labeled as juveniles and were not given marks anterior to each hind limb, as we have observed the cutaneous layers in this region are too thin to reliably hold marks in place. *Desmognathus* salamanders are not consistantly sexually dimorphic, and determining sex of most individuals requires sacrificing the animal for internal inspection of the gonads. However, when possible during the breeding season we recorded the sex of adult salamanders based on secondary sexual characters including mental gland and papillose vent in males and plicate vent in females (Noble, 1931); limited sample size precluded incorporation of sex into growth models. Salamanders were returned to their exact capture location within ~2 days after capture, but occasionally, salamanders were held up to 5 days to avoid releasing during unusually high flow events. For more details on sampling, see Halloran et al. (2021). All research was conducted with approval by the University of Arkansas Institutional Animal Care and Use Committee (AUP 14032).

### 2.5 Data analysis

#### 2.5.1 Body size

We examined variation in body size of recently metamorphed salamanders (i.e., less than a year since metamorphosis, ≤45 mm SVL) from June of each year at control and treatment sites with box plots. We then compared body condition of salamanders at control and treatment sites with a one‐way ANOVA on ranked residuals (Welsh et al., 2008) where the log of mass (g) depended on the following predictors: log of snout‐vent length (mm) and treatment (i.e., log (Mass) ~ log (SVL) + treatment). Salamanders included in the body condition analysis are approximately the same subset of those included in the growth analysis (below) but excluded gravid females. All analyses and figures were constructed in RStudio using R version 3.6.0 (R Core Team, 2021). Model assumption was verified by plotting residuals versus fitted values.

#### 2.5.2 Growth

We assessed growth of post‐metamorphic *D*. *brimleyorum* with Wang's (1998) parameterization of the Fabens method for estimating von Bertalanffy growth model parameters. The Wang (1998) model is parameterized in terms of the growth increment, *Z*, as:

$$Z=\left[l_{\infty }+\beta \left\{X‐E(X)\right\}‐X\right]\left(1‐e^{‐kT}\right)+\varepsilon$$

where *Z* is the change in salamander SVL; *l_∞_* + *β*{*X*–*E*(*X*)} is a first‐order approximation to the asymptotic SVL (Wang, 1998), where *l_∞_* (the population mean asymptotic length) and *β* are parameters to be estimated, *X* is the salamander SVL at the beginning of the interval, and *E*(*X*) is the sample mean SVL; *k* is the growth coefficient; *T* is the interval between recaptures; and *ε* is a term for model and measurement error. The final dataset excluded captures <21 days apart (within primary sampling intervals), as we assumed growth to be negligible relative to the resolution of our measurements within this time interval. We expanded the Wang (1998) model by including log‐normal random intercepts for site and year to account for spatial and temporal differences in *k*. Because of sparse data in some site‐by‐year combinations, we truncated the random site and year effects to be ±2, corresponding to a sevenfold increase or decrease relative to the mean growth rate for a given site or year. We further expanded the model to estimate the effect of harvest on log(*k*) using a binary indicator for harvest (0 = pre‐harvest or control, 1 = post‐harvest). The submodel for *k* was therefore:

$\log\left(k_{i}\right)=\alpha_{0}+\alpha \times {\text{treatment}}_{i}+\eta_{\text{site}\;i}+\eta_{\text{year}\;i},\;\text{where}$

$\eta_{\text{site}\;i}∼\text{Gaussian}\left(0,\sigma_{\text{site}}\right),\;\text{and}$

$\eta_{\text{year}\;i}∼\text{Gaussian}\left(0,\sigma_{\text{year}}\right)$

We used vague priors for all model parameters: uniform(min. = 0, max = 0.1) for *k*, Gaussian(mean = 0, SD = 10) for the log‐scale effect of treatment on *k*, Gaussian(0, 1) for *β*, uniform(0, 100) for *l*_∞_, and exponential(*λ* = 1) for all standard deviations. For growth intervals that spanned both pre‐and post‐harvest conditions, we specified the harvest covariate as missing and gave the missing data a Bernoulli(probability = 0.5) prior. Similarly, for intervals that spanned multiple years, we integrated model results over the interval by drawing the year effect from a categorical distribution with equal probability given to each year spanned by the interval between captures. To assess model fit, we used a posterior predictive check by simulating data under the model and calculating a Bayesian *p*‐value using sum of squares for the observed and simulated data (Kéry, 2010).

We implemented the model in a Bayesian framework using the software Just Another Gibbs Sampler version 4.3.0 (JAGS; Plummer, 2015) as called from R version 3.5.1 (R Core Team, 2021) using the package “jagsUI” (Kellner, 2016). We sampled from the posterior distribution using five independent chains of 1,000,000 iterations each after a burn‐in period of 200,000 iterations, and thinned chains by a factor of 50 to base inference on 100,000 samples from the posterior distribution. We used the Gelman and Rubin statistic (Gelman & Rubin, 1992) and examination of history plots to assess convergence; we observed no evidence for lack of convergence (all $\hat{R}<1.03$ and history plots appeared well‐mixed with no trends). Unless indicated otherwise, posterior distributions are summarized as median (0.025 quantile – 0.975 quantile).

## 3 RESULTS

**FIGURE 2:**
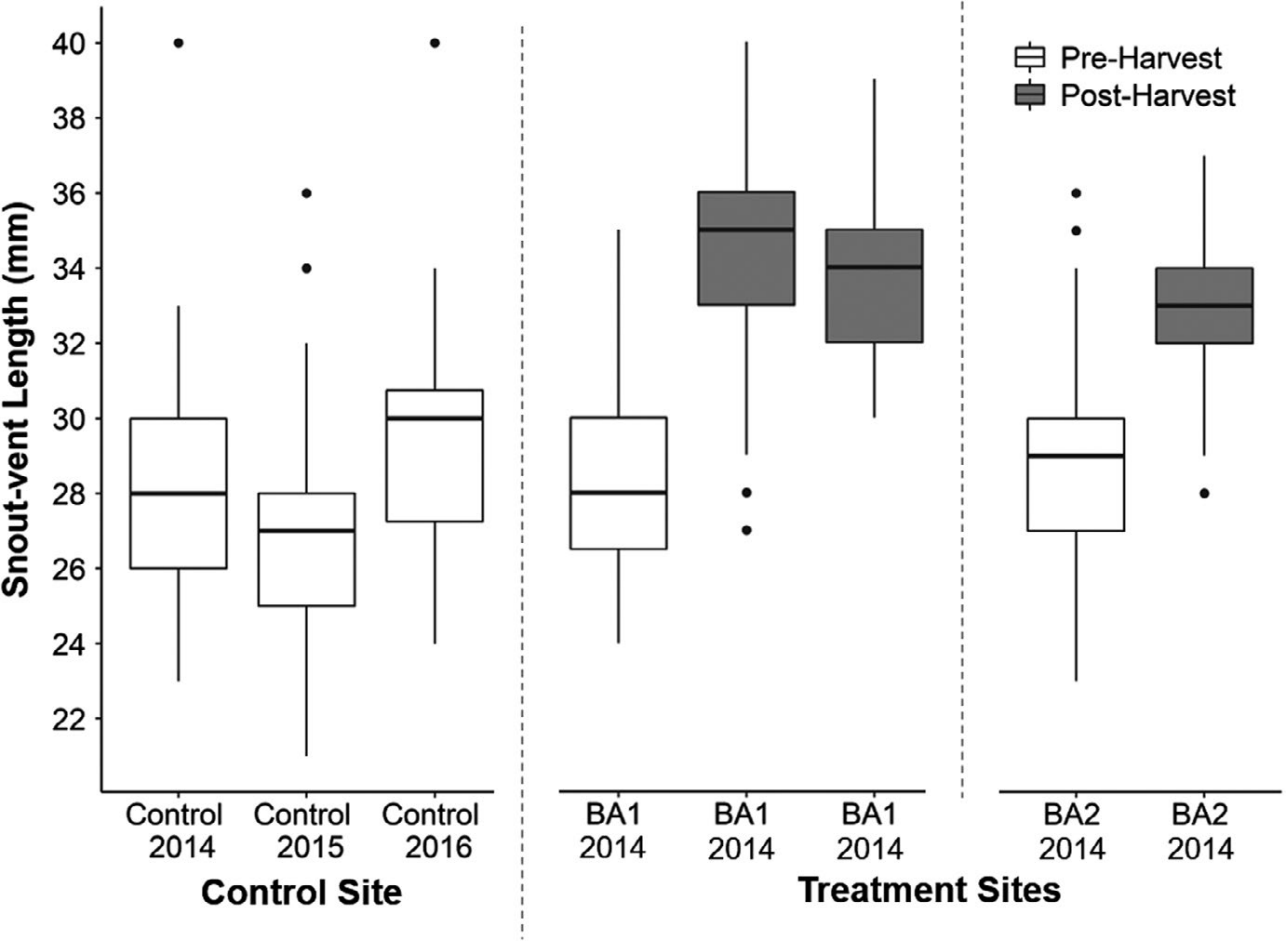
Pre‐ and post‐harvest mean body size (snout–vent length) for recently metamorphosed (i.e., less than a year since metamorphosis, ≤45 mm SVL) *Desmognathus brimleyorum* captured in June each year at control and treatment sites. In each box plot, the horizontal bar is the median, boxes correspond to the first and third quartiles, and whiskers extend to the highest value within 1.5*interquartile range; data beyond the whiskers are plotted as points

**FIGURE 3:**
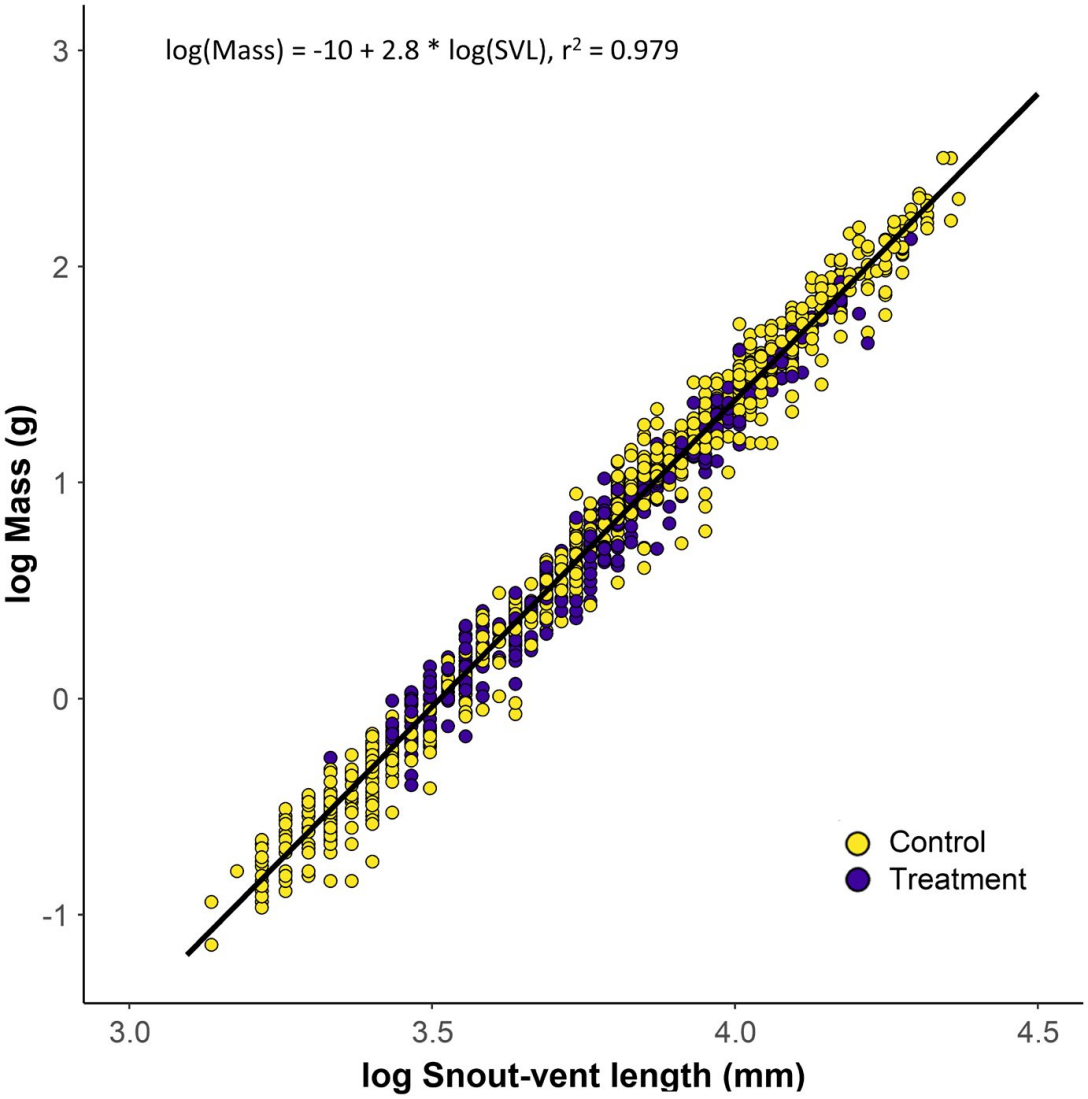
Relationship between the log of snout–vent length and log of mass for *Desmognathus brimleyorum* for control (unharvested and pre‐harvest; yellow circles) and treatment (post‐harvest; indigo circles) sites

We measured and marked 1,509 individual *D*. *brimleyorum* during this study. A subset of 839 individuals (control, *n* = 342; BA1, *n* = 135; BA2, *n* = 362) were recaptured between 1 and 6 times for a total of 1,229 captures (by site: control, *n* = 571; BA1, *n* = 178; BA2, *n* = 480; by year: 2014, *n* = 86; 2015, *n* = 533; 2016, *n* = 610), and these data were used to estimate growth before and after clear‐cut timber harvest. Based on raw data for June of each year, shortly after metamorphosis, mean body sizes of recently metamorphosed *D*. *brimleyorum* were greater in all three post‐harvest site–years than in the five pre‐harvest/control site–years (Figure 2). On average, salamanders were 5.7 and 4.0 mm larger post‐harvest at BA1 and BA2, respectively (Figure 2). Our body condition analysis included 1,103 captures (control = 738, treatment = 368), and body condition was not influenced by treatment (Figure 3; *R* ^2^ = 0.20, *F*_1,1103_ = 2.99, *p* = .084).

**FIGURE 4:**
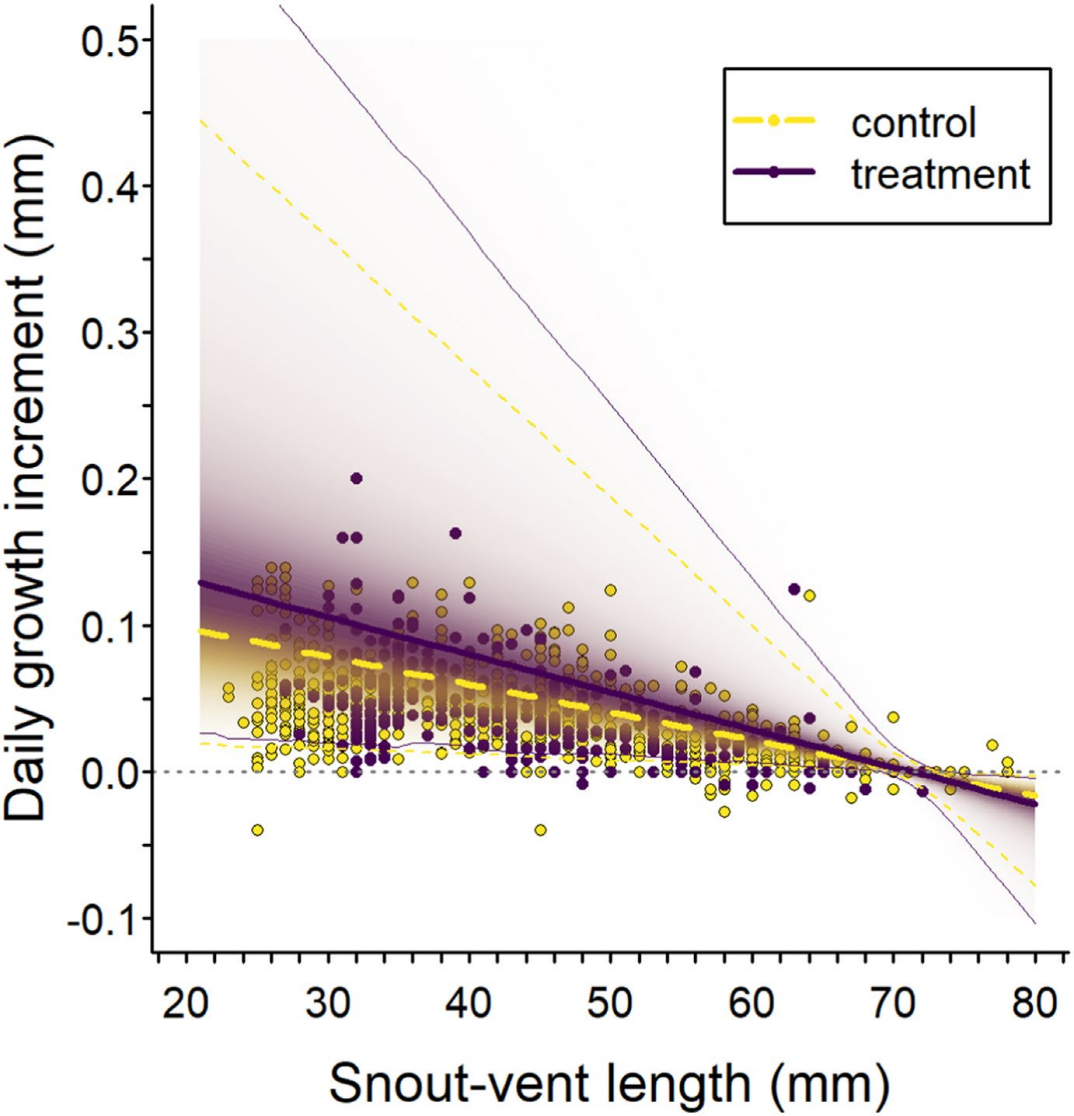
Relationship between growth increment and snout–vent length of *Desmognathus brimleyorum* under control (unharvested and pre‐harvest; yellow dashed lines and yellow circles) and treatment (post‐harvest; solid indigo lines and indigo circles) conditions. Bold lines represent posterior modes, narrow lines represent 95% highest posterior density intervals, the intensity of shading represents the posterior probability density, and points represent observed values. The horizontal gray dotted line along the *x*‐axis at zero represents a daily growth increment of zero

**FIGURE 5:**
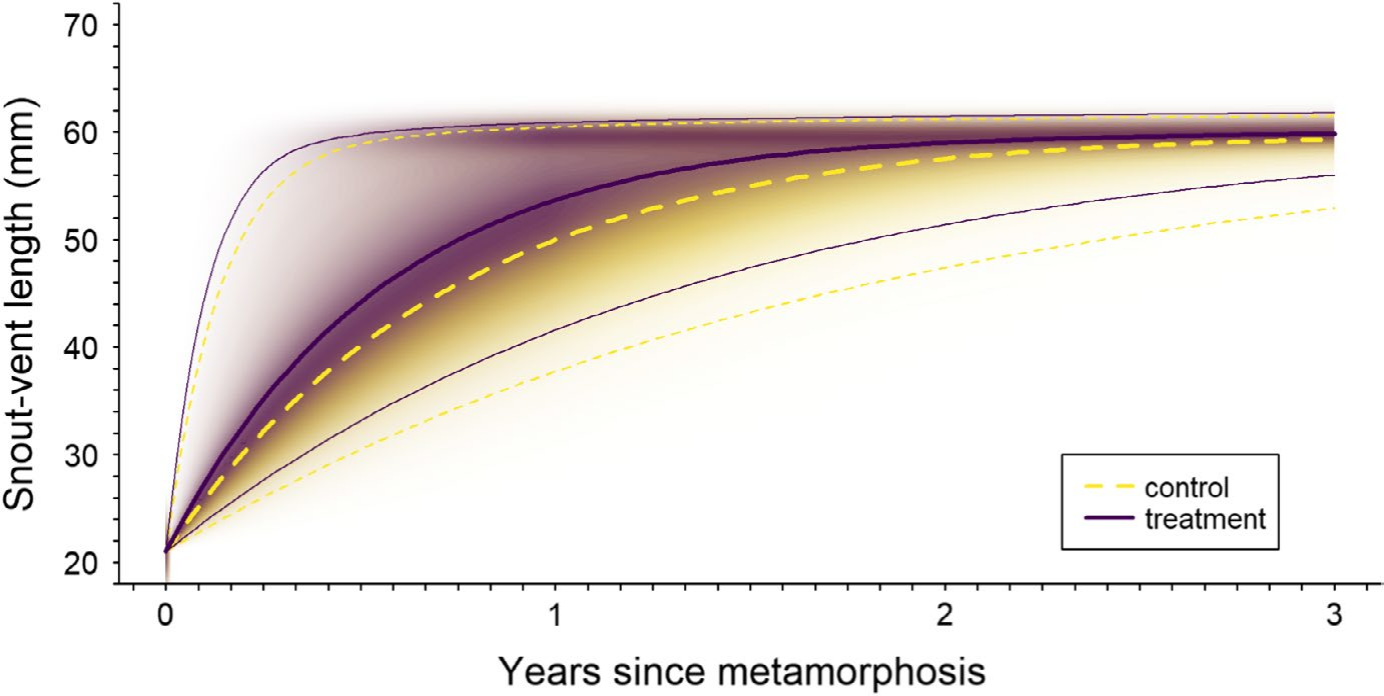
Population mean growth curves for *Desmognathus brimleyorum* under control (unharvested and pre‐harvest; dashed yellow lines) and treatment (post‐harvest; solid indigo lines) conditions. Bold lines represent posterior modes, light lines represent 95% highest posterior density intervals, and the intensity of shading represents the posterior probability density

The growth model fit our data well (Bayesian *p*‐value = .49). We found a positive effect of treatment (mean alpha = 0.29, 95% CRI −0.03 to 0.48) on salamander growth rate, *k*, with *k* 1.4 (95% credible interval 0.98–1.6) times greater following harvest in treated sites (posterior probability of a positive effect = 0.97; Figure 4). Variation in *k* was similar among sites (*σ*_site_ = 0.35 [0.12 to 2.58]) and years (*σ*_year_ =0.46 [0.16 to 3.05]). Model‐estimated mean asymptotic length among salamanders in this study was 60.3 (58.8–62.2)‐mm SVL (Figure 5). Model‐based expected individual asymptotic lengths ranged from 52 to 75 mm, with positive growth increments estimated for salamanders up to 73 mm SVL (*x*‐intercept of Figure 5).

## 4 DISCUSSION

We conducted a three‐year capture–mark–recapture study examining growth of 839 *D*. *brimleyorum*, before and after clear‐cut timber harvesting. Across both treatment sites and years, growth rates were 40% higher after harvest. Our study is among the first to examine stream salamander responses to timber harvesting at the individual level, an approach that is particularly notable as sublethal effects caused by harvesting may influence salamander population dynamics.

Increased growth rates of salamanders post‐harvest may influence *D*. *brimleyorum* populations in several ways. For *Desmognathus* species, body size and egg production are positively correlated, such that larger individuals are more fecund (Tilley, 1968), a common relationship in salamanders (Kaplan & Salthe, 1979; Salthe, 1969). Salamanders with faster growth rates could reach sexual maturity earlier or have higher fecundity at first reproduction, which may have significant effects on individual lifetime fitness and emergent population dynamics (Bernardo, 1993; Homyack & Haas, 2009; Tilley, 1977, 1980). Additionally, *Desmognathus* salamanders are territorial and prone to cannibalism, and salamander assemblages are often structured by competition and intraguild predation (Camp & Lee, 1996; Hairston, 1986). Thus, larger size may result in competitive and survival advantages over conspecifics. Finally, increased growth rates may reduce predation risk, as many important predators of salamanders in headwater streams (e.g., fish, frogs, snakes, other salamanders) are gape‐limited.

Several non‐mutually exclusive factors may explain increased growth of *D*. *brimleyorum* following harvest of the surrounding stand. In conjunction with faster growth rates post‐harvest (Figure 4), average body size (SVL) of newly metamorphosed salamanders in June was ~4–5 mm longer post‐harvest compared with pre‐harvest (Figure 2), suggesting variation in growth is initiated during the larval stage. Larval salamander growth rates can be affected by density of conspecifics and competition for resources (e.g., Morin et al., 1983; Petranka & Sih, 1986; Semlitsch, 1987). However, timber harvest did not have a negative effect on abundance or apparent survival of *D*. *brimleyorum* at either treatment site during our study (Halloran et al., 2021). Thus, release from intraspecific competition is not likely to be the primary mechanism driving the differences in growth rate we observed.

Alternatively, a likely mechanism driving increased growth rates may be a shift in the quality, quantity, or composition of invertebrate prey available. At our treatment sites, canopy cover decreased ~20% post‐harvest. Following overstory harvest, there is typically a temporary increase in light, stimulating primary production in streams (e.g., Webster et al., 1983) even among streams that retain riparian buffers (Kiffney et al., 2004). As a consequence, there is often an increase in productivity of grazer macroinvertebrate assemblages that feed on algal growth (Duncan & Brusven, 1985; Murphy, 1998; Murphy et al., 1981; Price et al., 2003; Silsbee & Larson, 1983; Wallace & Gurtz, 1986). In headwater streams, scraper and collector–gatherer functional feeding groups (i.e., biofilm consumers) are typically higher in nutrient content than are shredder taxa, although this pattern is highly variable (Cross et al., 2003; Frost et al., 2006). Importantly, algae are the primary source of fatty acids in aquatic food webs; thus, increases in biofilm consumers could lead to increased intake of essential fatty acids (Ballantyne et al., 2003; Brett & Muller‐Navarra, 1997), which are important for salamander growth (Fitzpatrick, 1976). Additionally, chitin content of macroinvertebrates varies (Cauchie, 2002), and prey may be assimilated differently depending on digestibility. Thus, it is possible that post‐harvest conditions favor production of invertebrate prey with increased assimilation (i.e., more energy dense and/or easier to digest), contributing to increased salamander growth rates.

Increased invertebrate prey quantity may also influence increased salamander growth rates post‐harvest. Studies have shown that macroinvertebrate abundance and density increase immediately post‐harvest (Haggerty et al., 2004; Kiffney et al., 2003; Wallace & Ely, 2014), even at sites with riparian buffers (Kiffney et al., 2003). Local resources influence growth in many populations, and greater prey availability increased salamander growth in several studies (Bernardo, 1994; Bernardo & Agosta, 2003; Tilley, 1974). For example, Bumpers et al. (2017) documented increased *Desmognathus quadramaculatus* growth due to increased abundance of invertebrate prey, because of experimental phosphorus enrichment of headwater streams. Following timber harvesting, the amount of dissolved nutrients leached from soil to the stream may increase until vegetation becomes reestablished, though riparian buffers minimize overland flow of water into streams (Swank, 1988; Swank et al., 2001; Webster et al., 1992). Fertilizer application to newly planted stands may further increase nutrient inputs to streams (Binkley et al., 1999), although riparian buffers can minimize inputs (Kastendick et al., 2012; Secoges et al., 2013). Similarly, disturbance to the upland overstory may add a brief pulse of nutrients in the form of organic matter to streams after harvest. Thus, it is likely that nutrient enrichment was at least partially responsible for the effects we observed and there is potential for this effect to be magnified under management regimes that include fertilization of newly planted timber stands.

Importantly, although we observed faster growth rates of salamanders post‐harvest, possibly attributed to shifts in availability of invertebrate prey, we did not find evidence of a larger body condition (i.e., mass relative to body length) after harvest, and thus, energy stores were similar within two years post‐harvest. Conversely, studies of terrestrial salamanders in managed forests of the Pacific Northwest and Eastern United States have found reduced body condition in recently harvested forests (Homyack et al., 2011; Welsh et al., 2008). Small sample sizes of previous studies in conjunction with a focus on terrestrial species (Homyack et al., 2011; Welsh et al., 2008) complicate comparisons across forest management regimes, particularly because harvested streams in our study had riparian buffers.

Because salamanders are ectothermic, a possible mechanism driving increased growth rates post‐harvest may be changes to metabolism because of increased air and water temperatures. In laboratory experiments, salamanders grow faster under warmer conditions (Beachy, 1995), and recent work has suggested a link between warming climate and increased salamander body sizes (McCarthy et al., 2017). Numerous studies have established that harvesting of riparian vegetation increases stream temperature (Herunter et al., 2004; Johnson & Jones, 2000; Wilkerson et al., 2006). However, the magnitude of stream temperature response to harvest may vary with inclusion of riparian buffers. Riparian buffers at our treatment sites were 14 and 21 m wide, and studies of streams in British Columbia with similar buffer widths documented 1–4°C increases in stream temperatures following harvest of the surrounding stand (Herunter et al., 2004; Kiffney et al., 2003; Macdonald et al., 2003). At streams with riparian buffers, increased stream temperatures following harvest have been observed to persist for 5 years (Macdonald et al., 2003). It is important to consider the potential biological consequences of even small changes in thermal regime, as temperature influences nearly every aspect of the physiology of ectotherms, including salamanders (Rome et al., 1992). Additionally, warmer temperatures can influence seasonal activity of *Desmognathus* salamanders (Shealy, 1975), resulting in a slightly extended activity season during cooler months, which may increase juvenile salamander growth rates following harvest.

## 5 CONCLUSIONS

Our intensive capture–recapture study of >800 individuals contributes to improved understanding of effects of timber harvesting on stream salamanders. Compared with control/pre‐harvest sites, we documented higher post‐harvest growth rates of *D*. *brimleyorum* at two treatment sites up to two years post‐harvest. Our study did not measure stream and air temperatures or prey availability and consumption before and after harvest; therefore, we are limited in our ability to identify mechanisms responsible for these patterns. However, given that timber harvesting did not affect salamander abundance or apparent survival (Halloran et al., 2021), a release from intraspecific competition is not a likely mechanism. Alternatively, juvenile salamanders may have different behavioral responses to harvesting or may capitalize on post‐harvest resource pulses resulting from a shift in the quality, quantity, or composition of invertebrate prey available. Additionally, changes to metabolism from potentially increased air and water temperatures post‐harvest may favor increased growth or result in a slightly extended activity season during cooler months. Addressing these potential mechanisms was beyond the scope of this study, and low replication (i.e., two treatment sites) reduces our ability to determine how robust our findings are across managed forests. However, our results suggest that harvesting may have short‐term positive effects on growth as has been seen with fish (e.g., Wilzbach et al., 2005), and thus may reduce predation risk or offset potential negative sublethal effects associated with harvest. However, this conclusion is predicated on the fact that ~20 m riparian buffers (i.e., SMZs) were retained along each side of our streams during harvest. Forestry BMPs for our study region recommend minimum buffers of 11–24 m (Arkansas Forestry Commission, 2002) to protect water quality (Cristan et al., 2016). To develop more focused and efficient management approaches, future studies may seek to determine the mechanistic relationships driving changes in growth rates post‐harvest, which could include monitoring shifts in the abiotic environment, invertebrate community, and salamander bioenergetics before and after harvest.

## CONFLICT OF INTEREST

None declared.

## AUTHOR CONTRIBUTIONS

**Jacquelyn C. Guzy:** Conceptualization (equal); data curation (lead); formal analysis (supporting); investigation (lead); methodology (lead); project administration (lead); visualization (equal); writing—original draft (lead); writing—review and editing (lead). **Brian J. Halstead:** Conceptualization (equal); data curation (equal); formal analysis (lead); visualization (equal); writing—original draft (supporting); writing—review and editing (equal). **Kelly M. Halloran:** Conceptualization (equal); data curation (equal); investigation (equal); methodology (equal); writing–review and editing (equal). **Jessica A. Homyack:** Conceptualization (equal); funding acquisition (equal); resources (equal); writing–original draft (supporting); writing–review and editing (equal). **John D. Willson:** Conceptualization (equal); funding acquisition (equal); investigation (equal); project administration (equal); resources (equal); writing—original draft (supporting); writing—review and editing (supporting).

## Data Availability

Salamander growth data and model code from this study can be accessed from the Dryad Digital Repository at https://doi.org/10.5061/dryad.2fqz612q3.

## References

[ece38238-bib-0001] Arkansas Forestry Commission, A . (2002). Best management practices for water quality protection.

[ece38238-bib-0002] Ash, A. N. (1997). Disappearance and return of Plethodontid Salamanders to clearcut plots in the Southern Blue Ridge Mountains. Conservation Biology, 11, 983–989.

[ece38238-bib-0003] Ballantyne, A. P. , Brett, M. T. , & Schindler, D. E. (2003). The importance of dietary phosphorus and highly unsaturated fatty acids for sockeye (*Oncorhynchus nerka*) growth in Lake Washington a bioenergetics approach. Canadian Journal of Fisheries and Aquatic Sciences, 60, 12–22.

[ece38238-bib-0004] Beachy, C. K. (1995). Effects of larval growth history on metamorphosis in a stream‐dwelling salamander (*Desmognathus ochrophaeus*). Journal of Herpetology, 29, 375–382. 10.2307/1564987

[ece38238-bib-0005] Bernardo, J. (1993). Determinants of maturation in animals. Trends in Ecology & Evolution, 8, 166–173. 10.1016/0169-5347(93)90142-C 21236138

[ece38238-bib-0006] Bernardo, J. (1994). Experimental analysis of allocation in two divergent, natural salamander populations. The American Naturalist, 143, 14–38. 10.1086/285594

[ece38238-bib-0007] Bernardo, J. , & Agosta, S. J. (2003). Determinants of clinal variation in life history of dusky salamanders (*Desmognathus ocoee*): Prey abundance and ecological limits on foraging time restrict opportunities for larval growth. Journal of Zoology, 259, 411–421. 10.1017/S0952836903003406

[ece38238-bib-0008] Binkley, D. , Burnham, H. , & Allen, H. L. (1999). Water quality impacts of forest fertilization with nitrogen and phosphorus. Forest Ecology and Management, 121, 191–213. 10.1016/S0378-1127(98)00549-0

[ece38238-bib-0009] Brett, M. , & Muller‐Navarra, D. (1997). The role of highly unsaturated fatty acids in aquatic foodweb processes. Freshwater Biology, 38(3), 483–499. 10.1046/j.1365-2427.1997.00220.x

[ece38238-bib-0010] Bumpers, P. M. , Rosemond, A. D. , Maerz, J. C. , & Benstead, J. P. (2017). Experimental nutrient enrichment of forest streams increases energy flow to predators along greener food‐web pathways. Freshwater Biology, 62, 1794–1805. 10.1111/fwb.12992

[ece38238-bib-0011] Camp, C. D. , & Lee, T. P. (1996). Intraspecific spacing and interaction within a population of *Desmognathus quadramaculatus* . Copeia, 1996, 78–84. 10.2307/1446943

[ece38238-bib-0012] Cauchie, H.‐M. (2002). Chitin production by arthropods in the hydrosphere. Hydrobiologia, 470, 63–95.

[ece38238-bib-0013] Cecala, K. K. , Lowe, W. H. , & Maerz, J. C. (2014). Riparian disturbance restricts in‐stream movement of salamanders. Freshwater Biology, 59, 2354–2364. 10.1111/fwb.12439

[ece38238-bib-0014] Cecala, K. K. , Price, S. J. , & Dorcas, M. E. (2007). A comparison of the effectiveness of recommended doses of MS‐222 (tricaine methanesulfonate) and Orajel^®^(benzocaine) for amphibian anesthesia. Herpetological Review, 38, 63.

[ece38238-bib-0015] Chazal, A. C. , & Niewiarowski, P. H. (1998). Responses of mole salamanders to clearcutting: Using field experiments in forest management. Ecological Applications, 8, 1133–1143.

[ece38238-bib-0016] Connette, G. M. , & Semlitsch, R. D. (2015). A multistate mark–recapture approach to estimating survival of PIT‐tagged salamanders following timber harvest. Journal of Applied Ecology, 52, 1316–1324. 10.1111/1365-2664.12472

[ece38238-bib-0017] Cristan, R. , Aust, W. M. , Bolding, M. C. , Barrett, S. M. , Munsell, J. F. , & Schilling, E. (2016). Effectiveness of forestry best management practices in the United States: Literature review. Forest Ecology and Management, 360, 133–151. 10.1016/j.foreco.2015.10.025

[ece38238-bib-0018] Cross, W. F. , Benstead, J. P. , Rosemond, A. D. , & Bruce Wallace, J. (2003). Consumer‐resource stoichiometry in detritus‐based streams. Ecology Letters, 6, 721–732. 10.1046/j.1461-0248.2003.00481.x

[ece38238-bib-0019] Davic, R. D. , & Welsh, H. H. (2004). On the ecological roles of salamanders. Annual Review of Ecology Evolution and Systematics, 35, 405–434. 10.1146/annurev.ecolsys.35.112202.130116

[ece38238-bib-0020] Duncan, W. F. , & Brusven, M. A. (1985). Benthic macroinvertebrates in logged and unlogged low‐order southeast Alaskan streams. Freshwater Invertebrate Biology, 4, 125–132. 10.2307/1467102

[ece38238-bib-0021] Fitzpatrick, L. C. (1976). Life history patterns of storage and utilization of lipids for energy in amphibians. American Zoologist, 16, 725–732. 10.1093/icb/16.4.725

[ece38238-bib-0022] Frost, P. C. , Benstead, J. P. , Cross, W. F. , Hillebrand, H. , Larson, J. H. , Xenopoulos, M. A. , & Yoshida, T. (2006). Threshold elemental ratios of carbon and phosphorus in aquatic consumers. Ecology Letters, 9, 774–779. 10.1111/j.1461-0248.2006.00919.x 16796566

[ece38238-bib-0024] Gelman, A. , & Rubin, D. B. (1992). Inference from iterative simulation using multiple sequences. Statistical Science, 7, 457–472. 10.1214/ss/1177011136

[ece38238-bib-0025] Grant, E. H. C. (2008). Visual implant elastomer mark retention through metamorphosis in amphibian larvae. Journal of Wildlife Management, 72, 1247–1252. 10.2193/2007-183

[ece38238-bib-0026] Gurtz, M. E. , & Wallace, J. B. (1984). Substrate‐mediated response of stream invertebrates to disturbance. Ecology, 65, 1556–1569. 10.2307/1939135

[ece38238-bib-0027] Guzy, J. C. , Halloran, K. M. , Homyack, J. A. , & Willson, J. D. (2019). Influence of riparian buffers and habitat characteristics on salamander assemblages in headwater streams within managed forests. Forest Ecology and Management, 432, 868–883. 10.1016/j.foreco.2018.10.006

[ece38238-bib-0028] Haggerty, S. , Batzer, D. , & Jackson, C. (2004). Macroinvertebrate response to logging in coastal headwater streams of Washington, USA. Canadian Journal of Fisheries and Aquatic Sciences, 61, 529–537. 10.1139/f04-014

[ece38238-bib-0029] Hairston, N. G. Sr (1986). Species packing in *Desmognathus* salamanders: Experimental demonstration of predation and competition. American Naturalist, 127(3), 266–291. 10.1086/284485

[ece38238-bib-0030] Halloran, K. M. , Guzy, J. C. , Homyack, J. A. , & Wilson, J. D. (2021). Effects of timber harvest on survival and movement of stream salamanders in a managed forest landscape. Ecosphere, 12(4), e03489.

[ece38238-bib-0031] Herbeck, L. A. , & Larsen, D. R. (1999). Plethodontid salamander response to silvicultural practices in Missouri Ozark forests. Conservation Biology, 13, 623–632. 10.1046/j.1523-1739.1999.98097.x

[ece38238-bib-0032] Hernández‐Pacheco, R. , Plard, F. , Grayson, K. L. , & Steiner, U. K. (2021). Demographic consequences of changing body size in a terrestrial salamander. Ecology and Evolution, 11(1), 174–185. 33437421 10.1002/ece3.6988 PMC7790640

[ece38238-bib-0033] Herunter, H. , MacDonald, J. , & MacIsaac, E. (2004). Effectiveness of variable‐retention riparian buffers for maintaining thermal regimes, water chemistry, and benthic invertebrate communities of small headwater streams in central British Columbia. In Forest Land‐fish Conference II—Ecosystem Stewardship through Collaboration, Proc. Forest‐Land‐FishConf. II (pp. 105–113).

[ece38238-bib-0034] Hocking, D. J. , Connette, G. M. , Conner, C. A. , Scheffers, B. R. , Pittman, S. E. , Peterman, W. E. , & Semlitsch, R. D. (2013). Effects of experimental forest management on a terrestrial, woodland salamander in Missouri. Forest Ecology and Management, 287, 32–39. 10.1016/j.foreco.2012.09.013

[ece38238-bib-0035] Homyack, J. A. , & Haas, C. A. (2009). Long‐term effects of experimental forest harvesting on abundance and reproductive demography of terrestrial salamanders. Biological Conservation, 142(1), 110–121.

[ece38238-bib-0036] Homyack, J. A. , Haas, C. A. , & Hopkins, W. A. (2011). Energetics of surface‐active terrestrial salamanders in experimentally harvested forest. The Journal of Wildlife Management, 75, 1267–1278. 10.1002/jwmg.175

[ece38238-bib-0037] Huntsman, B. M. , Venarsky, M. P. , Benstead, J. P. , & Huryn, A. D. (2011). Effects of organic matter availability on the life history and production of a top vertebrate predator (Plethodontidae: *Gyrinophilus palleucus*) in two cave streams. Freshwater Biology, 56, 1746–1760. 10.1111/j.1365-2427.2011.02609.x

[ece38238-bib-0038] Johnson, B. R. , & Wallace, J. B. (2005). Bottom‐up limitation of a stream salamander in a detritus‐based food web. Canadian Journal of Fisheries and Aquatic Sciences, 62, 301–311. 10.1139/f04-197

[ece38238-bib-0039] Johnson, S. L. , & Jones, J. A. (2000). Stream temperature responses to forest harvest and debris flows in western Cascades, Oregon. Canadian Journal of Fisheries and Aquatic Sciences, 57, 30–39. 10.1139/f00-109

[ece38238-bib-0040] Johnston, B. , & Frid, L. (2002). Clearcut logging restricts the movements of terrestrial Pacific giant salamanders (*Dicamptodon tenebrosus* Good). Canadian Journal of Zoology, 80, 2170–2177.

[ece38238-bib-0041] Kaplan, R. H. , & Salthe, S. N. (1979). The allometry of reproduction: an empirical view in salamanders. The American Naturalist, 113, 671–689. 10.1086/283425

[ece38238-bib-0042] Karraker, N. , & Welsh, H. (2006). Long‐term impacts of even‐aged timber management on abundance and body condition of terrestrial amphibians in Northwestern California. Biological Conservation, 131, 132–140. 10.1016/j.biocon.2006.02.013

[ece38238-bib-0043] Kastendick, D. N. , Zenner, E. K. , Palik, B. J. , Kolka, R. K. , & Blinn, C. R. (2012). Effects of harvesting on nitrogen and phosphorus availability in riparian management zone soils in Minnesota, USA. Canadian Journal of Forest Research, 42, 1784–1791. 10.1139/x2012-127

[ece38238-bib-0044] Kellner, K. (2016). jagsUI: A Wrapper Around ‘rjags’ to Streamline ‘JAGS’Analyses. Version 1.4. 2.

[ece38238-bib-0045] Kéry, M. (2010). Introduction to WinBUGS for ecologists: Bayesian approach to regression, ANOVA, mixed models and related analyses. Academic Press.

[ece38238-bib-0046] Kiffney, P. M. , Richardson, J. S. , & Bull, J. P. (2003). Responses of periphyton and insects to experimental manipulation of riparian buffer width along forest streams. Journal of Applied Ecology, 40, 1060–1076. 10.1111/j.1365-2664.2003.00855.x

[ece38238-bib-0047] Kiffney, P. M. , Richardson, J. S. , & Bull, J. P. (2004). Establishing light as a causal mechanism structuring stream communities in response to experimental manipulation of riparian buffer width. Journal of the North American Benthological Society, 23(3), 542–555.

[ece38238-bib-0048] Kroll, A. J. , Risenhoover, K. , McBride, T. , Beach, E. , Kernohan, B. J. , Light, J. , & Bach, J. (2008). Factors influencing stream occupancy and detection probability parameters of stream‐associated amphibians in commercial forests of Oregon and Washington, USA. Forest Ecology and Management, 255(11), 3726–3735. 10.1016/j.foreco.2008.03.005

[ece38238-bib-0049] Lee, P. , Smyth, C. , & Boutin, S. (2004). Quantitative review of riparian buffer width guidelines from Canada and the United States. Journal of Environmental Management, 70, 165–180. 10.1016/j.jenvman.2003.11.009 15160742

[ece38238-bib-0101] Lemmon, P. E. (1956). A spherical densiometer for estimating forest overstory density. Forest Science, 2, 314–320. 10.1093/forestscience/2.4.314

[ece38238-bib-0050] Lowe, R. L. , Golladay, S. W. , & Webster, J. R. (1986). Periphyton response to nutrient manipulation in streams draining clearcut and forested watersheds. Journal of the North American Benthological Society, 5, 221–229. 10.2307/1467709

[ece38238-bib-0051] Macdonald, J. , MacIsaac, E. , & Herunter, H. (2003). The effect of variable‐retention riparian buffer zones on water temperatures in small headwater streams in sub‐boreal forest ecosystems of British Columbia. Canadian Journal of Forest Research, 33, 1371–1382. 10.1139/x03-015

[ece38238-bib-0052] Maigret, T. A. , Cox, J. J. , Schneider, D. R. , Barton, C. D. , Price, S. J. , & Larkin, J. L. (2014). Effects of timber harvest within streamside management zones on salamander populations in ephemeral streams of southeastern Kentucky. Forest Ecology and Management, 324, 46–51. 10.1016/j.foreco.2014.03.043

[ece38238-bib-0053] McCarthy, T. , Masson, P. , Thieme, A. , Leimgruber, P. , & Gratwicke, B. (2017). The relationship between climate and adult body size in redback salamanders (*Plethodon cinereus*). Geo: Geography and Environment, 4(1), e00031.

[ece38238-bib-0054] Means, D. B. (1974). The status of Desmognathus brimleyorum Stejneger and an analysis of the genus *Desmognathus* (Amphibia: Urodela) in Florida. Bulletin of the Florida State Museum, Biological Science, 18(1). https://ufdc.ufl.edu/UF00000975/00001

[ece38238-bib-0055] Means, D. B. (1975). Evolutionary ecology studies on salamanders of the genus Desmognathus. Part I: Competitive exclusion along a habitat gradient between two species of salamanders (Desmognathus) in western Florida; Part II: Life history, growth and body size variation in populations of a streamside salamander (*Desmognathus brimleyorum*) on adjacent mountains. Ph.D. dissertation. Florida State University, Tallahassee, Florida.

[ece38238-bib-0102] Means, D. B. (1999). Desmognathus brimleyorum. Catalogue of American Amphibians and Reptiles (CAAR). http://hdl.handle.net/2152/45473

[ece38238-bib-0057] Morin, P. J. , Wilbur, H. M. , & Harris, R. N. (1983). Salamander predation and the structure of experimental communities: responses of *Notophthalmus* and microcrustacea. Ecology, 64, 1430–1436. 10.2307/1937497

[ece38238-bib-0058] Murphy, M. L. (1998). Primary production. In R. J. Naiman & R. E. Bilby (Eds.), River ecology and management: Lessons from the Pacific Coastal Ecoregion (pp. 144–168). Springer‐Verlag.

[ece38238-bib-0059] Murphy, M. L. , Hawkins, C. P. , & Anderson, N. (1981). Effects of canopy modification and accumulated sediment on stream communities. Transactions of the American Fisheries Society, 110, 469–478.

[ece38238-bib-0060] Noble, G. K. (1931). The biology of the Amphibia. Dover Publications.

[ece38238-bib-0061] Olson, D. H. , Anderson, P. D. , Frissell, C. A. , Welsh, H. H. Jr , & Bradford, D. F. (2007). Biodiversity management approaches for stream–riparian areas: perspectives for Pacific Northwest headwater forests, microclimates, and amphibians. Forest Ecology and Management, 246, 81–107. 10.1016/j.foreco.2007.03.053

[ece38238-bib-0062] Perkins, D. W. , & Hunter, M. L. (2006). Use of amphibians to define riparian zones of headwater streams. Canadian Journal of Forest Research, 36, 2124–2130. 10.1139/x06-111

[ece38238-bib-0063] Peterman, W. E. , Crawford, J. A. , & Semlitsch, R. D. (2008). Productivity and significance of headwater streams: population structure and biomass of the black‐bellied salamander (*Desmognathus quadramaculatus*). Freshwater Biology, 53, 347–357.

[ece38238-bib-0064] Peterman, W. E. , & Semlitsch, R. D. (2009). Efficacy of riparian buffers in mitigating local population declines and the effects of even‐aged timber harvest on larval salamanders. Forest Ecology and Management, 257, 8–14. 10.1016/j.foreco.2008.08.011

[ece38238-bib-0104] Petranka, J. W. (1998). Salamanders of the United States and Canada. Smithsonian Institution Press.

[ece38238-bib-0065] Petranka, J. W. , Brannon, M. P. , Hopey, M. E. , & Smith, C. K. (1994). Effects of timber harvesting on low elevation populations of southern Appalachian salamanders. Forest Ecology and Management, 67, 135–147. 10.1016/0378-1127(94)90012-4

[ece38238-bib-0066] Petranka, J. W. , & Sih, A. (1986). Environmental instability, competition, and density‐dependent growth and survivorship of a stream‐dwelling salamander. Ecology, 67, 729–736. 10.2307/1937696

[ece38238-bib-0067] Plummer, M. (2015). JAGS version 4.0. 0 user manual. See https://sourceforge.net/projects/mcmc‐jags/files/Manuals/4

[ece38238-bib-0068] Price, K. , Suski, A. , McGarvie, J. , Beasley, B. , & Richardson, J. S. (2003). Communities of aquatic insects of old‐growth and clearcut coastal headwater streams of varying flow persistence. Canadian Journal of Forest Research, 33, 1416–1432. 10.1139/x03-089

[ece38238-bib-0069] R Core Team (2021). R: A language and environment for statistical computing. R Foundation for Statistical Computing. https://www.R‐project.org/

[ece38238-bib-0070] Reichenbach, N. , & Sattler, P. (2007). Effects of timbering on *Plethodon hubrichti* over twelve years. Journal of Herpetology, 41, 622–629. 10.1670/06-170.1

[ece38238-bib-0071] Reiter, M. , Bilby, R. E. , Beech, S. , & Heffner, J. (2015). Stream temperature patterns over 35 years in a managed forest of western Washington. JAWRA Journal of the American Water Resources Association, 51(5), 1418–1435.

[ece38238-bib-0072] Rome, L. , Stevens, E. D. , & John‐Alder, H. (1992). The influence of temperature and thermal acclimation on physiological function. In M. E. Feder & W. W. Burggren (Eds.), Environmental physiology of the amphibians (pp. 183–205). The University of Chicago Press.

[ece38238-bib-0073] Salthe, S. N. (1969). Reproductive modes and the number and sizes of ova in the urodeles. American Midland Naturalist, 81(2), 467–490. 10.2307/2423983

[ece38238-bib-0074] Secoges, J. M. , Aust, W. M. , Seiler, J. R. , Dolloff, C. A. , & Lakel, W. A. (2013). Streamside management zones affect movement of silvicultural nitrogen and phosphorus fertilizers to Piedmont streams. Southern Journal of Applied Forestry 37, 26–35. 10.5849/sjaf.11-032

[ece38238-bib-0075] Semlitsch, R. D. (1987). Density‐dependent growth and fecundity in the paedomorphic salamander *Ambystoma talpoideum* . Ecology, 68, 1003–1008. 10.2307/1938371

[ece38238-bib-0076] Shealy, R. M. (1975). Factors influencing activity in the salamanders *Desmognathus ochrophaeus* and *D. monticola* (Plethodontidae). Herpetologica, 31, 94–102.

[ece38238-bib-0077] Silsbee, D. G. , & Larson, G. L. (1983). A comparison of streams in logged and unlogged areas of Great Smoky Mountains National Park. Hydrobiologia, 102, 99–111. 10.1007/BF00006073

[ece38238-bib-0078] Southerland, M. T. , Jung, R. E. , Baxter, D. P. , Chellman, I. C. , Mercurio, G. , & Vølstad, J. H. (2004). Stream salamanders as indicators of stream quality in Maryland, USA. Applied Herpetology, 2, 23–46. 10.1163/1570754041231596

[ece38238-bib-0079] Swank, W. T. (1988). Stream chemistry responses to disturbance. In W. T. Swank & D. A. Crossley Jr. (Eds.), Forest Hydrology and Ecology at Coweeta (pp. 339–357). Springer. https://link.springer.com/chapter/10.1007/978‐1‐4612‐3732‐7_25

[ece38238-bib-0080] Swank, W. T. , Vose, J. , & Elliott, K. (2001). Long‐term hydrologic and water quality responses following commercial clearcutting of mixed hardwoods on a southern Appalachian catchment. Forest Ecology and Management, 143, 163–178. 10.1016/S0378-1127(00)00515-6

[ece38238-bib-0082] Tilley, S. G. (1968). Size‐fecundity relationships and their evolutionary implications in five desmognathine salamanders. Evolution, 22, 806–816. 10.1111/j.1558-5646.1968.tb03479.x 28562853

[ece38238-bib-0083] Tilley, S. G. (1974). Structures and dynamics of populations of the salamander *Desmognathus ochrophaeus* Cope in different habitats. Ecology, 55, 808–817.

[ece38238-bib-0084] Tilley, S. G. (1977). Studies of life histories and reproduction in North American plethodontid salamanders. In The reproductive biology of amphibians (pp. 1–41). Springer.

[ece38238-bib-0085] Tilley, S. G. (1980). Life histories and comparative demography of two salamander populations. Copeia, 1980(4), 806–821. 10.2307/1444460

[ece38238-bib-0103] Trauth, S. E. (1988). Egg clutches of the Ouachita Dusky Salamander, *Desmognathus brimleyorum* (Caudata: Plethodontidae), collected in Arkansas during a summer drought. In S. E. Trauth (Ed.), The Southwestern Naturalist, (Vol. 33, No. 2, pp. 234–236). Southwestern Association of Naturalists. 10.2307/3671902

[ece38238-bib-0086] Trauth, S. E. , Cox, R. L., Jr , Butterfield, B. P. , Saugey, D. A. , & Meshaka, W. E. Jr (1990). Reproductive phenophases and clutch characteristics of selected Arkansas amphibians. Journal of the Arkansas Academy of Science, 44, 107–113.

[ece38238-bib-0088] Walker, D. M. , Murray, C. M. , Talbert, D. , Tinker, P. , Graham, S. P. , & Crowther, T. W. (2018). A salamander's top down effect on fungal communities in a detritivore ecosystem. FEMS Microbiology Ecology, 94(12), fiy168. 10.1093/femsec/fiy168 30247565

[ece38238-bib-0089] Wallace, J. (1988). Aquatic invertebrate research. In W. T. Swank & D. A. Crossley Jr. (Eds.), Forest hydrology and ecology at Coweeta, (Vol. 66, pp. 257–268). Springer‐Verlag. 10.1007/978-1-4612-3732-7_19

[ece38238-bib-0090] Wallace, J. B. , Eggert, S. L. , Meyer, J. L. , & Webster, J. R. (1997). Multiple trophic levels of a forest stream linked to terrestrial litter inputs. Science, 277, 102–104. 10.1126/science.277.5322.102

[ece38238-bib-0091] Wallace, J. B. , & Ely, D. (2014). Stream macroinvertebrate response to clearcut logging. In W. T. Swank & J. R. Webster (Eds.), Long‐term response of a forest watershed ecosystem: Clearcutting in the Southern Appalachians (pp. 177–193). Oxford University Press. 10.1093/acprof:osobl/9780195370157.003.0014

[ece38238-bib-0092] Wallace, J. B. , & Gurtz, M. E. (1986). Response of Baetis mayflies (Ephemeroptera) to catchment logging. American Midland Naturalist, 115(1), 25–41. 10.2307/2425834

[ece38238-bib-0093] Wang, Y. (1998). An improved Fabens method for estimation of growth parameters in the von Bertalanffy model with individual asymptotes. Canadian Journal of Fisheries and Aquatic Sciences, 55(2), 397–400. 10.1139/f97-211

[ece38238-bib-0094] Webster, J. , Golladay, S. , Benfield, E. , Meyer, J. , Swank, W. , & Wallace, J. (1992). Catchment disturbance and stream response: an overview of stream research at Coweeta Hydrologic Laboratory. In P. J. Boon , P. Calow , & G. E. Petts (Eds.), River Conservation and Management, 15, 232–253. John Wiley & Sons.

[ece38238-bib-0095] Webster, J. , Gurtz, M. , Hains, J. , Meyer, J. , Swank, W. , Waide, J. , & Wallace, J. (1983). Stability of stream ecosystems. In J. R. Barnes & G. Wayne Minshall (Eds.), Stream ecology (pp. 355–395). Springer. 10.1007/978-1-4613-3775-1_14

[ece38238-bib-0096] Webster, J. R. , & Waide, J. B. (1982). Effects of forest clearcutting on leaf breakdown in a southern Appalachian stream. Freshwater Biology, 12, 331–344. 10.1111/j.1365-2427.1982.tb00627.x

[ece38238-bib-0097] Welsh, H. H. , Pope, K. L. , & Wheeler, C. A. (2008). Using multiple metrics to assess the effects of forest succession on population status: A comparative study of two terrestrial salamanders in the US Pacific Northwest. Biological Conservation, 141(4), 1149–1160. 10.1016/j.biocon.2008.02.014

[ece38238-bib-0098] Wilkerson, E. , Hagan, J. M. , Siegel, D. , & Whitman, A. A. (2006). The effectiveness of different buffer widths for protecting headwater stream temperature in Maine. Forest Science, 52, 221–231.

[ece38238-bib-0099] Wilzbach, M. A. , Harvey, B. C. , White, J. L. , & Nakamoto, R. J. (2005). Effects of riparian canopy opening and salmon carcass addition on the abundance and growth of resident salmonids. Canadian Journal of Fisheries and Aquatic Sciences, 62(1), 58–67.

[ece38238-bib-0100] Wyman, R. L. (1998). Experimental assessment of salamanders as predators of detrital food webs: effects on invertebrates, decomposition and the carbon cycle. Biodiversity and Conservation, 7, 641–650.

